# Relationship between Children’s Birth Weight and Birth Length and a Risk of Overweight and Obesity in 4–15-Year-Old Children

**DOI:** 10.3390/medicina55080487

**Published:** 2019-08-15

**Authors:** Joanna Baran, Aneta Weres, Ewelina Czenczek-Lewandowska, Justyna Leszczak, Katarzyna Kalandyk-Osinko, Artur Mazur

**Affiliations:** 1 Institute of Physiotherapy, Medical Faculty, University of Rzeszów, 35-959 Rzeszów, Poland; 2 Institute of Midwifery and Medical Emergency, Medical Faculty, University of Rzeszów, 35-959 Rzeszów, Poland; 3 Institute of Nursing and Health Sciences, Medical Faculty, University of Rzeszów, 35-959 Rzeszów, Poland

**Keywords:** body birth weight, body birth length, pediatric obesity, pediatric overweight

## Abstract

*Background and Objectives*. The purpose of the study was to investigate the relationship between children’s birth weight/length and a risk of overweight and obesity. *Materials and Methods*. The study involved 747 children from kindergartens, as well as primary and middle schools from southeastern Poland. All the subjects were examined on fasting status. Each child was examined for body mass and height, in order to calculate their body mass index (BMI), and BMI centile. The parents completed a questionnaire related to basic information about the child and the family. *Results*. In the study group, the male infants presented greater birth body weight and birth body length. A comparison of the distribution of birth weights and lengths between the children with normal BMI and with high BMI showed statistically significant differences only in the case of birth length of 12–15-year-old children and in the group of boys aged 12–15 years. In the case of the female children and the group of 7–11-year-olds a statistically significant difference was found in the BMI centile at a later age—a higher centile was found in the girls and in the children aged 7–11 years classified as adequate for gestational age (AGA). *Conclusions*. Birth body weight is positively related to BMI centile; however, no significant differences were found in birth weight between children with overweight/obesity and children with normal body weight. Birth length is associated with a lower BMI centile only in boys aged 12–15 years, and lower birth length is found in boys with overweight and obesity.

## 1. Introduction

The normal course of pregnancy may be related to childhood overweight or obesity. Important factors determining the probability that a child will be affected by overweight or obesity include, e.g., his/her birth weight and length. A positive linear relationship between birth weight and obesity was observed by many researchers. The related findings show, e.g., that each increase in birth weight by 100 g is associated with a greater likelihood of obesity [1,2].

There are sex-related differences in the relationship between birth body weight and risk of overweight and obesity in children. Excessive birth weight is linked with a greater risk of overweight or obesity both in girls and in boys. On the other hand, low birth weight is associated with a lower risk of overweight and obesity only in girls. These differences should be taken into account in the process of designing interventions aimed at reducing overweight and obesity in children [3]. Furthermore, a high birth weight, particularly very high, was shown to increase the likelihood of type 2 diabetes in young male adults [4]. 

In the mechanism of catch-up growth, children with low birth weight postnatally show compensatory growth in accordance with their genetic determinants and then rapidly increase body weight.

A postnatal growth spike and risk of obesity may occur when offspring that experienced a protein deficiency are exposed to an energy-rich diet. Several auto-, para-, and endocrine hormonal factors stimulating adipose tissue growth can induce adipose tissue expansion. Among them, insulin-like growth factor 2 (IGF2), which is produced by one of the best known epigenetically imprinted genes, is associated with higher body weight and obesity. One possible mechanism for increasing IGF2 expression in adipose tissue is increased methylation of differentially methylated regions (DMR) in the H19 control region (ICR/H19) [5].

Recent studies showed that prenatal low-protein intake and postnatal high-fat intake lead to an increase in adipose tissue through changes in *IGF2* gene expression and CpG IGF2/H19 methylation in the tissue itself. These studies confirmed that histone methylation may result in different transcription consequences depending on the effect of the residues [6]. A study by Chinese researchers showed that both very high and very low birth weight are risk factors for overweight and obesity in older children. Moderately high birth weight was also associated with a greater risk of central obesity [7].

Based on birth weight and/or birth length, as well as timing of delivery relative to the expected due date, it can be determined whether or not the birth weight and/or birth length are adequate for gestational age (AGA). It was established, based on available evidence, that birth weight and length appropriate for gestational age is in the range between the 10th and 90th centiles, relative to the gestational age. The related values below the 10th centile and above the 90th centile correspond to children rated as small for gestational age (SGA) and large for gestational age (LGA), respectively [8].

Both large and small birth weight may predispose in the future, inter alia, to metabolic and cardiovascular diseases. Some authors argued that the threshold values for SGA are reflected by the fifth, third, or even second centiles [9,10].

It is sometimes assumed that SGA is a synonym for intrauterine growth restriction (IUGR), a condition reflected by low body weight and/or length of the fetus and/or neonate, relative to the expected values. Therefore, IUGR is always a pathology, while the population of children classified as SGA at birth also includes healthy, constitutionally small children [11]. IUGR may be identified when two intrauterine measurements of the fetus show that the growth rate is too slow and/or when the neonate’s birth weight or length is less than −2 SD (or <10th centile) in relation to gestational age [12].

Intrauterine growth restriction is associated with complications which may develop at every stage of life. For instance, a susceptibility to impaired glucose tolerance and type 2 diabetes occur in childhood, while adolescence is linked with a predisposition to early puberty, and adulthood is additionally associated with a greater risk of a metabolic syndrome [13].

The purpose of the study was to investigate the relationship between children’s birth weight/length and a risk of overweight and obesity.

## 2. Materials and Methods

### 2.1. Participants

The kindergartens and schools were randomly selected for the study. Invitations were sent to 1000 families with children in educational institutions in southeastern Poland. Replies were received from 858 families. The eligibility criteria included consent of the parent or legal guardian for their child’s participation in the study, the child’s consent to participate, and the child’s age in the range 3–18 years. The study group did not include children failing to meet the above eligibility criteria, or children with disabilities or lower limb injuries adversely affecting the children’s vertical position. Overall, 57 parents did not agree to participate in the study, 12 children did not agree to the study, 10 children were not fasting on the day of the study, 15 children were absent on the day of the study, and 17 mothers did not provide data on the course of pregnancy. Finally, the study involved 747 mothers and children. After obtaining consent, it turned out that the age range of respondents was within the range of 4–15 years. The subjects’ mean age was 9.36 years ± 3.52 years. The girls and the boys did not differ significantly in terms of age.

Parents were informed about the objectives and course of the examinations, and about the criteria excluding children from the study. All the subjects were examined on fasting status, and consecutive measurements were performed in the morning. Each child was examined for body mass and height, in order to calculate their body mass index (BMI) and BMI centile.

The parents completed a questionnaire related to basic information about the child and the family, and provided copies of the child’s health record and personal maternity record.

According to demographic data, in the area where the research was carried out in 2017, 2,129,138 residents lived there and 51% of them were women and 49% were men. Furthermore, 14.4% of the female population were girls and 15.8% of the male population were boys of pre-school and school ages. Due to the fact that the occurrence of excessive body weight in children shows a large discrepancy with regard to sex, the sample size was calculated separately for girls and boys. The size of the required sample was calculated, taking into account a 95% confidence level and the estimated average occurrence of the phenomenon of excessive body weight at the level of 20% in girls and 26% in boys. The level of significance was considered as *p* < 0.05. It was calculated that the minimum sample size should be 246 girls and 296 boys.

### 2.2. Anthropometric Measurements

Body height was measured with a Seca 213 stadiometer (Seca GmbH & Co. KG., Hamburg, Germany). The subject was standing barefoot with his/her back to the measuring rod of the stadiometer. Body height was measured three times, and the mean value was calculated to eliminate measurement error. The appliance conforms with Annex VI to Directive 93/42/EEC concerning medical devices.

Body mass was assessed using Tanita BC 420 MA, an analyzer complying with applicable requirements, in accordance with European standards and directives, i.e., NAWI (Non-Automatic Weighing Instruments) and MDD (Medical Device Directive). Devices carrying the MA symbol are designed for medical applications, as they are validated and approved for such use. The analyzer was certified for compliance with 93/42 EEC (the European Union (EU) standard for medical devices).

### 2.3. Assessment of Perinatal Risk Factors

Information related to perinatal risk factors for overweight and obesity was retrieved from the child’s health record and personal maternity record. The relevant data included the child’s birth body weight and birth body length, as well as duration of pregnancy.

After the above data were collected, the children’s current BMI centiles were calculated by reference to the Polish centile grids [14], and the children’s body weight categories were determined based on the BMI centiles, and by reference to the classification proposed by Barlow et al. [15].

Based on their body weight at birth, the children were classified as hypertrophic neonates (body weight over 4000 g—macrosomia), eutrophic neonates (body weight in the range of 2500–4000 g—in multiple births, the lower limit is 2200 g), neonates with low birth weight (LBW, <2500 g), neonates with very low birth weight (VLBW, <1500 g), neonates with extremely low birth weight (ELBW, <1000 g), and neonates with incredibly low birth weight (ILBW, <750 g), by reference to the Centers for Disease Control (CDC) classification [16].

The children were also classified based on their body weight at birth relative to the duration of gestation: adequate for gestational age (AGA—birth body weight in the range between the 10th and 90th centile, relative to the gestational age), small for gestational age (SGA—birth body weight below the 10th centile, relative to the gestational age), and large for gestational age (LGA—birth body weight over the 90th centile, relative to the gestational age) [17].

### 2.4. Data Analysis

Data analysis was carried out using selected methods of descriptive statistics and statistical inference. Selected numerical characteristics of the tested parameters were determined: number (*n*), percentage (%), *x* (mean), Me (median), and standard deviation (s).

To determine the level of statistical significance, chi-square independence tests and Mann–Whitney nonparametric tests (for comparing two groups) or Kruskal–Wallis tests (for comparing more than three groups) were used. The statistical significance level was *p* < 0.05.

For the correlation of variables that did not meet the criterion of the normality of distribution, the Spearman rank correlation coefficient was calculated.

The analysis also used logistic regression using the input method to verify the effect of interfering factors on test results.

Calculations were performed with Statistica 10.0 (StatSoft, Inc., Tulsa, OK, USA).

### 2.5. Ethical

The approval of the Bioethics Committee of the University of Rzeszów No. 18/12/2015 from 2 December 2015 was obtained for conducting the research.

## 3. Results

Analyses were conducted to examine the relationship of birth weight and birth length to abnormalities in BMI occurring at a later age. At birth, the children’s mean length was 55 ± 3.3 cm and mean weight was 3359 ± 565 g. In the study group, the male infants presented greater birth body weight (3430 g vs. 3281 g, *p* < 0.001 ***) and birth body length (55.3 cm vs. 54.8 cm, *p* = 0.049 *).

A comparison of the distribution of birth weights and lengths between the children with normal BMI and with high BMI showed statistically significant differences only in the case of birth length of 12–15-year-old children (*p* = 0.048 *) and in the group of boys aged 12–15 years (*p* = 0.003 **) (Table 1).

Additionally, an alternative analysis was conducted to examine a BMI centile-based classification of the children in relation to their birth weight and length. This analysis also took into account the children’s age and sex, since the impact of perinatal factors may be greater in the case of younger children.

The findings presented below show that there were statistically significant correlations between birth weight and BMI centile in the entire study group (*p* = 0.046 *), in the entire group of girls (*p* = 0.028 *), in the group of girls aged 7–11 years (*p* = 0.008 *), and in the two younger age groups (*p* = 0.027 * for the children aged 4–6 and *p* = 0.029 * for the children aged 7–11 years). Greater birth weights correspond to higher BMI centiles. Additionally, one negative correlation was found—in the group of boys aged 12–15 years. Greater birth length coincided with the subject’s lower BMI centile (*p* = 0.039 *) (Table 2).

Birth weights were classified based on defined norms, and according to norms relative to gestational age. Analysis of the association between centile-based classification of birth weight and the BMI centile at a later age was performed relative to sex and age; however, the LGA type was disregarded (due to the very small size of this group—only seven subjects). In the case of the female children and the group of 7–11-year-olds, a statistically significant difference was found in the BMI centile at a later age—a higher centile was found in the girls (*p* = 0.035 *) and in the children aged 7–11 years (*p* = 0.009 **) classified as AGA (Table 3).

Subsequent classification was related to the children’s birth weights, which in a majority of the cases were in the range of 2500–4000 g (84.5%). Statistically significant differences were found between the girls and the boys (*p* < 0.001 ***). The chi-square test of independence showed a sex-related difference, namely, more frequent occurrence of hypertrophic and less frequent occurrence of eutrophic type among male neonates. In further analyses, the LBW group included the children with birth weight classified as VLBW and ILBW.

Analysis of the study group relative to hypertrophic, eutrophic, and low birth weight showed that the children with greater birth weight also presented a higher value of the current BMI centile. A decrease in these values corresponded to lower birth weight, yet the relationship was not statistically significant. Sex- and age-related differences in BMI centile relative to birth weight classification were also insignificant (Table 3).

Overweight or obesity at a later age is equally common, irrespective of the type of birth weight classification. A comparison of the second birth weight classification to the BMI classification shows a relationship approaching statistical significance (the *p*-value was only slightly higher than 0.05). The LBW group comprised relatively more children with low body mass, and fewer children with normal BMI relative to age and sex (Table 4).

The effects of birth weight centile classification were also examined in relation to overweight/obesity occurring in the children. No statistically significant differences in overweight/obesity rates occurring at a later age were identified in the children representing the specific groups (Table 5).

The relationship between birth weight and birth length and the occurrence of overweight/obesity was not burdened with disturbing factors such as age, sex, socioeconomic status, and parents’ education, and their mutual influence on the child’s body weight status was checked. Logistic regression analysis showed that no disturbing variable significantly differentiated the effect of birth weight and birth length on the prevalence of overweight/obesity in children (*p* > 0.05) (Table 6).

## 4. Discussion

Numerous studies provide evidence supporting the hypothesis that the size of the child at birth is related to a risk of diseases later in life. Such associations are well established, in particular for low birthweight and a greater risk of such conditions as coronary heart disease, diabetes, hypertension, and stroke in adulthood. These relationships are modified by growth patterns following birth [18]. Numerous researchers reported that high birth weight (>4000 g) was associated with a greater risk of obesity compared to birth weight <4000 g. Low birth weight (<2500 g) was related to a decreased risk of obesity. Analyses taking into account subgroups representing various stages of growth and development (preschoolers, school-age children, and adolescents) also demonstrated that high birth weight was associated with a greater risk of obesity from childhood to early adulthood.

The results suggest that people with low birth weight face decreased long-term risk of being overweight, while high birth weight predisposes for being overweight at an older age. Therefore, preventing excessive food intake, i.e., avoiding maternal overnutrition, overweight, and/or diabetes during pregnancy, may be a promising strategy for overweight prevention [19,20]. The above studies are consistent with the results of our own research, in which a higher value of birth weight correlates with a higher value BMI percentile of studies.

The longitudinal study of Lindberg et al. presented no significant differences between children with normal or low birth weight with respect to the prevalence of overweight/obesity; however, at the age of 3.5 years, the mean body height, body weight, and BMI in the children with marginally low birth weight were lower compared to the children in the control group [21].

An opposite trend was shown by Jornayvaz et al. [22], where middle-aged adults, born with low body weight, tended to more frequently present diabetes and obesity, as well as higher leptin level and leptin-to-body-fat ratio, compared to adults born with normal body weight.

The research review carried out by Ribeiro et al. suggests that both cardiometabolic events and obesity are linked to intrauterine nutritional deficiency, which, in combination with food supply exceeding the organism’s metabolic needs during early life stages, leads to endocrine changes [23].

According to a comprehensive literature review carried out by Araújo de França et al. [24], birth body weight seems to be associated with a generally larger body size in adulthood, including greater waist and hip circumference.

Children who are born small for gestational age (SGA) are more likely to develop obesity, insulin resistance, and type 2 diabetes [25]. Furthermore, in the case of children born small for gestational age, intrauterine nutritional deficiency may result in modified metabolic systems, adjusted to the expected chronic undernutrition. Potentially, these children are not prepared well to cope with high-calorie diets, and they may be programmed for storing greater amounts of energy, which leads to later obesity, metabolic syndromes, and impaired regulation of normal puberty, as well as early onset of cardiovascular diseases. In most cases, disturbed energy balance is a joint effect of genetics, epigenetics, and environmental factors [26].

It is estimated that, worldwide, 8–26% of neonates are born with low weight (LBW). These infants face a risk of medical problems during childhood and in adulthood. Scientific evidence suggests that the postnatal period is of critical importance, because lifelong predispositions to metabolic disorders and obesity may result from nutritional style experienced at this time. In view of the fact that optimum nutrition in the case of LBW, pre-term, and SGA infants continues to be a matter of dispute, it can be assumed that many infants are not managed in an optimal manner. This may lead to a rapid increase in body weight and continuous health problems [27,28].

It was also shown that the children born small for gestational age (SGA) presented lower BMI centile corresponding to their current body weight. This association was found in the girls and children aged 7–11 years.

A difference approaching statistical significance was also shown in the case of current body weight classification (based on BMI centile) versus birth weight classification. The highest percentage of the children with overweight and obesity had a birth weight below 2500 g (*p* = 0.069).

Research carried out on Croatian children of school age showed that the children with overweight and obesity were also found with slightly higher birth length compared to the children with normal body weight. A significant sex-related difference was found in the birth length, showing the boys’ advantage in this respect. Median birth lengths in the boys and in the girls were 51 cm (interquartile range (IQR): 49–52) and 50 cm (IQR: 49–52), respectively [29].

The present study shows an opposite relationship. The children with normal body weight at 12–15 years of age had greater birth length compared to the children with overweight and obesity. This relationship was confirmed by a negative correlation between birth length and current body weight centile among the oldest boys.

## 5. Conclusions

Birth body weight is positively related to BMI centile; however, no significant differences were found in birth weight between children with overweight/obesity and children with normal body weight. Being born small for gestational age is not linked with a greater risk and prevalence of overweight and obesity.

Birth length is associated with a lower BMI centile only in boys aged 12–15 years, and lower birth length is found in boys with overweight and obesity.

**Strengths and Limitations of the Study**

In future studies, it would be necessary to enlarge the number of examined children, especially for children aged 16–18, after puberty.

A limitation of this study is also the analysis of only two factors potentially affecting the occurrence of overweight and obesity in children, as well as the fact that the perinatal data came from medical records and not from a direct examination carried out by the authors.

In subsequent analyses, it would also be worth taking into account the influence of environmental or genetic factors, which would undoubtedly increase the scientific level of the paper. The study’s strength is that the sample is representative of the study population. In addition, comparable results were obtained with other researchers, which can be considered a general trend for children in the analyzed age group.

## Figures and Tables

**Table 1:** Relationship between children’s current body weight category and their birth weight and length.

Variables	Total			4–6 Years			7–11 Years			12–15 Years		
	Birth Body Weight (g)	Birth Body Length (cm)	Gestational Age (weeks)	Birth Body Weight (g)	Birth Body Length (cm)	Gestational Age (weeks)	Birth Body Weight (g)	Birth Body Length (cm)	Gestational Age (weeks)	Birth Body Weight (g)	Birth Body Length (cm)	Gestational Age (weeks)
Total												
Normal or lower body mass	3366	55.1	39.41	3266	54.6	39.10	3398	55.2	39.49	3398	55.4	39.55
Overweight or obesity	3317	54.8	39.43	3318	54.6	39.23	3349	55.2	39.43	3278	54.4	39.57
*p*	0.387	0.193	0.867	0.678	0.705	0.701	0.827	0.893	0.822	0.120	0.048	0.884
Boys												
Normal or lower body mass	3448	55.4	39.41	3278	54.8	39.08	3490	55.5	39.53	3506	55.7	39.49
Overweight or obesity	3328	54.5	39.29	3334	55.1	39.28	3294	54.6	39.15	3386	53.6	39.57
*p*	0.154	0.102	0.694	0.730	0.760	0.643	0.239	0.517	0.549	0.190	0.003	0.799
Girls												
Normal or lower body mass	3276	54.7	39.40	3256	54.4	39.12	3282	54.7	39.43	3286	55.0	39.61
Overweight or obesity	3305	55.0	39.59	3283	53.5	39.12	3416	55.9	39.78	3224	54.8	39.55
*p*	0.691	0.870	0.724	0.749	0.198	0.898	0.232	0.455	0.703	0.730	0.948	0.848

*p*—probability test value.

**Table 2:** Relationship between body mass index (BMI) centile and the children’s birth weight and length relative to age groups.

Infant’s Somatic Features	BMI Centile		
	Girls	Boys	Total
Total			
Birth body weight (g)	0.12 (0.028) ^a^	0.03 (0.520) ^a^	0.07 (0.046) ^a^
Birth body length (cm)	0.04 (0.431) ^a^	−0.01 (0.788) ^a^	0.02 (0.675) ^a^
4–6 years			
Birth body weight (g)	0.19 (0.072) ^a^	0.15 (0.160) ^a^	0.17 (0.027) ^a^
Birth body length (cm)	0.07 (0.488) ^a^	0.13 (0.208)	0.10 (0.173) ^a^
7–11 years			
Birth body weight (g)	0.21 (0.008) ^a^	0.04 (0.612) ^a^	0.12 (0.029) ^a^
Birth body length (cm)	0.08 (0.341) ^a^	0.01 (0.836) ^a^	0.05 (0.315) ^a^
12–15 years			
Birth body weight (g)	−0.02 (0.802) ^a^	−0.08 (0.410) ^a^	−0.08 (0.257) ^a^
Birth body length (cm)	−0.03 (0.766) ^a^	−0.21 (0.039) ^a^	−0.12 (0.090) ^a^

^a^—correlation with probability test value.

**Table 3:** Relationship between BMI centile and the classification of birthweight for gestational age in the children, relative to their sex and age.

Variables	Centile Classification of Birth Body Weight						
	SGA	AGA	*p*	Hypertrophic Type	Eutrophic Type	LBW Type	*p*
Sex							
Girls	30 ^a^	324 ^a^	0.035	19 ^a^	319 ^a^	18 ^a^	0.170
	36.5 (±32.0) ^b^	48.5 (±31.7) ^b^		53.6 (±30.1) ^b^	48.1 (±31.5) ^b^	36.3 (±37.2) ^b^	
Boys	25 ^a^	361 ^a^	0.242	56 ^a^	312 ^a^	23 ^a^	0.836
	40.7 (±26.8) ^b^	48.4 (±31.1) ^b^		48.8 (±30.6) ^b^	48.0 (±30.8) ^b^	43.6 (±31.1) ^b^	
Age							
4–6 years	17 ^a^	158 ^a^	0.747	12 ^a^	148 ^a^	18 ^a^	0.349
	45.3 (±26.3) ^b^	48.1 (±31.1) ^b^		54.5 (±26.5) ^b^	48.4 (±30.2) ^b^	38.8 (±35.1) ^b^	
7–11 years	27 ^a^	328 ^a^	0.009	48 ^a^	294 ^a^	17 ^a^	0.449
	30.0 (±29.2) ^b^	46.3 (±31.5) ^b^		48.6 (±32.1) ^b^	45.3 (±31.4) ^b^	36.9 (±30.7) ^b^	
12–15 years	11 ^a^	199 ^a^	0.691	15 ^a^	189 ^a^	6 ^a^	0.991
	48.3 (±31.8) ^b^	52.3 (±31.2) ^b^		51.2 (±28.9) ^b^	52.0 (±31.2) ^b^	55.3 (±39.3) ^b^	

^a^—number of subjects; ^b^—mean ± standard deviation; AGA—adequate for gestational age; LBW—low birth weight; *p*—probability test value; SGA—small for gestational age.

**Table 4:** The child’s body weight category-based BMI centile, relative to birth weight and gestational age.

Body Weight Category Based on BMI Centile	Underweight (*n* = 78)	Normal Weight (*n* = 552)	Overweight (*n* = 71)	Obesity (*n* = 46)	Total (*n* = 747)
Centile classification of birth weight (0.236) ^a^					
SGA	10 (18.2%)	40 (72.7%)	4 (7.3%) ^b^	1 (1.8%) ^b^	55
AGA	68 (9.9%)	505 (73.7%)	67 (9.8%) ^b^	45 (6.6%) ^b^	685
LGA	0 (0.0%)	7 (100.0%)	0 (0.0%) ^b^	0 (0.0%) ^b^	7
Birth weight classification (0.069) ^a^					
Hypertrophic	8 (10.7%)	59 (78.7%)	4 (5.3%)	4 (5.3%)	75
Eutrophic	60 (9.5%)	469 (74.3%)	62 (9.8%)	40 (6.4%)	631
LBW	10 (24.4%)	24 (58.5%)	5 (12.2%)	2 (4.9%)	41

^a^—probability test value; AGA—adequate for gestational age; ^b^—n (%); LBW—low birth weight; LGA—large for gestational weight; SGA—small for gestational age.

**Table 5:** Prevalence of overweight and obesity in children, relative to birth weight for gestational age.

Variables	Centile Classification of Birth Body Weight						
	SGA	AGA	*p*	Hypertrophic Type	Eutrophic Type	LBW Type	*p*
Sex							
Girls	2 (6.7%)	56 (17.3%)	0.133	3 (15.8%)	53 (16.6%)	2 (11.1%)	0.826
Boys	3 (12.0%)	56 (15.5%)	0.637	5 (8.9%)	49 (15.8%)	5 (21.7%)	0.279
Age							
4–6 years	1 (5.9%)	25 (15.8%)	0.273	1 (8.3%)	22 (14.9%)	3 (16.7%)	0.799
7–11 years	3 (11.1%)	46 (14.0%)	0.673	6 (12.5%)	40 (13.7%)	3 (17.6%)	0.868
12–15 years	1 (9.1%)	41 (20.6%)	0.353	1 (6.7%)	40 (21.2%)	1 (16.7%)	0.393

AGA—adequate for gestational age; LBW—low birth weight; LGA—large for gestational weight; *p*—probability test value; SGA—small for gestational age.

**Table 6:** Logistic regression of results.

Disturbing Variables		OR (95% CI)	*p*
Age			
4–6 years of age	Body birth weight	1.00 (1.00–1.00)	0.565
	Body birth length	0.96 (0.80–1.15)	0.670
7–11 years of age	Body birth weight	1.00 (1.00–1.00)	0.428
	Body birth length	1.04 (0.92–1.17)	0.546
12–15 years of age	Body birth weight	1.00 (1.00–1.00)	0.785
	Body birth length	0.88 (0.74–1.03)	0.116
Sex			
Male	Body birth weight	1.00 (1.00–1.00)	0.870
	Body birth length	0.93 (0.83–1.05)	0.269
Female	Body birth weight	1.00 (1.00–1.00)	0.998
	Body birth length	1.03 (0.91–1.16)	0.661
Maternal education			
Professional	Body birth weight	1.00 (1.00–1.01)	0.110
	Body birth length	0.77 (0.48–1.25)	0.294
Average	Body birth weight	1.00 (1.00–1.00)	0.704
	Body birth length	1.01 (0.89–1.14)	0.931
Higher	Body birth weight	1.00 (1.00–1.00)	0.523
	Body birth length	0.98 (0.87–1.11)	0.801
Father’s education			
Professional	Body birth weight	1.00 (1.00–1.00)	0.782
	Body birth length	1.01 (0.83–1.22)	0.946
Average	Body birth weight	1.00 (1.00–1.00)	0.145
	Body birth length	1.03 (0.91–1.17)	0.647
Higher	Body birth weight	1.00 (1.00–1.00)	0.437
	Body birth length	0.91 (0.79–1.05)	0.195
Socio-economic status			
Below average	Body birth weight	1.00 (1.00–1.00)	0.420
	Body birth length	0.94 (0.80–1.10)	0.418
Average	Body birth weight	1.00 (1.00–1.00)	0.072
	Body birth length	1.07 (0.95–1.21)	0.268
Above average	Body birth weight	1.00 (1.00–1.00)	0.099
	Body birth length	0.85 (0.70–1.02)	0.080

95% CI—95% confidence interval for the odds ratio; OR—odds ratio; *p*—level of statistical significance.
